# Driving Factors and Future Prediction of Land Use and Cover Change Based on Satellite Remote Sensing Data by the LCM Model: A Case Study from Gansu Province, China

**DOI:** 10.3390/s20102757

**Published:** 2020-05-12

**Authors:** Kongming Li, Mingming Feng, Asim Biswas, Haohai Su, Yalin Niu, Jianjun Cao

**Affiliations:** 1 College of Geography and Environmental Science, Northwest Normal University, Lanzhou 730070, China; 2018222416@nwnu.edu.cn (K.L.); 2018222392@nwnu.edu.cn (M.F.); 2019222458@nwnu.edu.cn (H.S.); 2019222440@nwnu.edu.cn (Y.N.); 2 School of Environmental Sciences, University of Guelph, 50 Stone Road East, Guelph, ON N1G 2W1, Canada; biswas@uoguelph.ca

**Keywords:** remote sensing, LUCC driving factors, land use prediction, logistic regression, CA-Markov model

## Abstract

Land use and cover change (LUCC) is an important issue affecting the global environment, climate change, and sustainable development. Detecting and predicting LUCC, a dynamic process, and its driving factors will help in formulating effective land use and planning policy suitable for local conditions, thus supporting local socioeconomic development and global environmental protection. In this study, taking Gansu Province as a case study example, we explored the LUCC pattern and its driving mechanism from 1980 to 2018, and predicted land use and cover in 2030 using the integrated LCM (Logistic-Cellular Automata-Markov chain) model and data from satellite remote sensing. The results suggest that the LUCC pattern was more reasonable in the second stage (2005 to 2018) compared with that in the first stage (1980 to 2005). This was because a large area of green lands was protected by ecological engineering in the second stage. From 1980 to 2018, in general, natural factors were the main force influencing changes in land use and cover in Gansu, while the effects of socioeconomic factors were not significant because of the slow development of economy. Landscape indices analysis indicated that predicted land use and cover in 2030 under the ecological protection scenario would be more favorable than under the historical trend scenario. Besides, results from the present study suggested that LUCC in arid and semiarid area could be well detected by the LCM model. This study would hopefully provide theoretical instructions for future land use planning and management, as well as a new methodology reference for LUCC analysis in arid and semiarid regions.

## 1. Introduction

Land use and cover has undergone great changes around the world over the past few decades [1], especially in developing countries with increasing populations and rapid urbanization [2]. Land use and cover change (LUCC) on earth’s land surface has been proven to be an essential driving factor for a series of regional and global environmental problems [3], such as carbon emission [4,5], climate change [6,7], biodiversity loss [8], ecosystem productivity decrease [9,10], soil and land degradation [11], as well as ecosystem services decline [12]. These environmental issues arouse people’s concerns about future developments, leading to the emergency of land changes science, which is regarded as the fundamental content of the global environment change and sustainability research [12,13]. On a global scale, the socioeconomic and political components were considered as the principal factors for LUCC [14]. Previous studies about LUCC mainly focused on its dynamic patterns [3,14], driving factors [15,16,17,18], effects on ecosystems [4,9,10], and dynamic simulation and prediction [14,19,20,21] at different spatiotemporal scales. It is widely thought that the driving mechanism analysis and spatiotemporal pattern prediction for LUCC in the future can help assess the direction and degree of changes in land use and cover, and are critical for sustainable land use and mitigation of global environmental problems regarding LUCC [13,22]. 

Cellular Automata-Markov chain (CA-Markov) model is a general method that has been widely used to predict LUCC in future scenarios, and mainly includes three parts: transition probability produced by Markov chain, transition rules defined by a CA model, and a collection of suitability maps [19,20]. In this model, the production of a suitability maps collection is most crucial and has a great effect on the definition of transition rule in CA and the accuracy of the final modeling results [19]. Previously, Multi-Criteria Evaluation (MCE), a multi-indicator decision-making method with three steps (i.e., indicator selection, parameter setting (score and weight of indicators), and constraint factors), was a common method for creating suitability maps [23,24]. However, in MCE, the indicator selection always depends on researcher’s subjective judgement and lack of mathematical analysis, and parameter setting is empirical and often influenced by the calculation method [19]. Therefore, this method is not linked to specific land use and cover changes and is generally a subjective and stochastic procedure to a large extent [19,25]. 

For improving the performance of the CA-Markov model, some mathematical statistics methods have been used to integrate it with the original CA-Markov, such as artificial neural networks [26], system dynamics [21], analytical hierarchy process [27], multilayer perceptron [28], random forest, as well as logistic regression model [29,30]. Among these models, the logistic regression method has been widely used with the traditional CA-Markov model (Logistic-CA-Markov model, LCM) due to its capacity to take the dynamic process of LUCC into consideration [20]. This is a generalized linear regression model that can well connect the categorical variables and the continuous variables and build potential relationships between them [31]. Cetin and Demirel [32] establish a preliminary framework of LCM for prediction of urban changes in the Istanbul metropolitan area. Fu et al. [19] explored the availability of an integrated LCM in predicting the future LUCC in Hamilton, OH, USA. He et al. [33] predicted the future land use and cover changes in the Beijing-Tianjin-Hebei metropolitan region using this model. Logistic regression models are also regarded as a reliable way of detecting the driving forces of LUCC and have been extensively applied in relative research. For instance, Arsanjani et al. [34] analyzed the driving factors of the suburban expansion in the metropolitan area of Tehran, Iran. Li et al. [16] detected the driving forces of urban expansion in Shenyang, China from 1997 to 2010. 

Arid and semiarid regions accounts for about 40% of earth’s land surface [35], which is generally ecologically fragile and vulnerable to environmental changes, most of them distributed in undeveloped areas, such as Northwest China and North Africa [14,36]. The LUCC in arid and semiarid region can not only affect local socioeconomic development and environment protection, but also influence global environmental changes [36]. Gansu Province is a typical arid and semiarid area constrained by a fragile ecological environment and belonging to the most impoverished area in China [37]. The objective of this study was to examine the LUCCs over the past 40 years, identify their dominant factors and predict future change in 2030, as predicted by LCM, taking Gansu Province, China as a case study example. This study aims to provide reasonable instructions for local land use policies and serve as a reference for the sustainable development in global arid and semiarid areas.

## 2. Materials and Methods

### 2.1. Study Area

**Figure 1:**
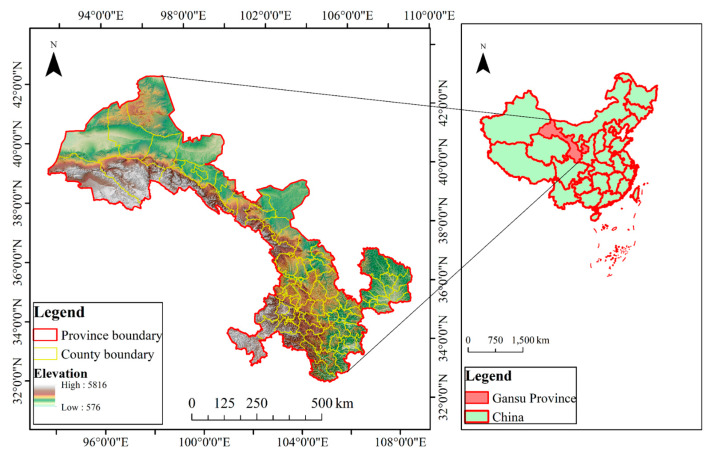
Location of Gansu Province.

Gansu Province (92°13′–108°46′E, 32°31′–42°57′N) is a typical arid and semi-arid region in northwest China (Figure 1), covering an area of about 4.56 × 10^5^ km^2^ [32]. Located in the intersection of the Qinghai–Tibet plateau, the Loess plateau, and the Inner Mongolian plateau, Gansu Province is a representative farming-pastoral zone and famous for its diversified natural and human landscapes. However, Gansu is also the most undeveloped area in China. Since 1970s, with the aim to protect the harsh natural environment, a series of environment protection policies have been implemented in this area, including the Three-North Forest Shelterbelts Program launched in 1979 and the Grain for Green Program (GGP) implemented in 1999 [4,38,39]. Complex natural and human background drove the land use and cover changes in Gansu, but also made it difficult to detect and predict the change process and its driving mechanisms.

### 2.2. Data Source and Processing 

**Figure 2:**
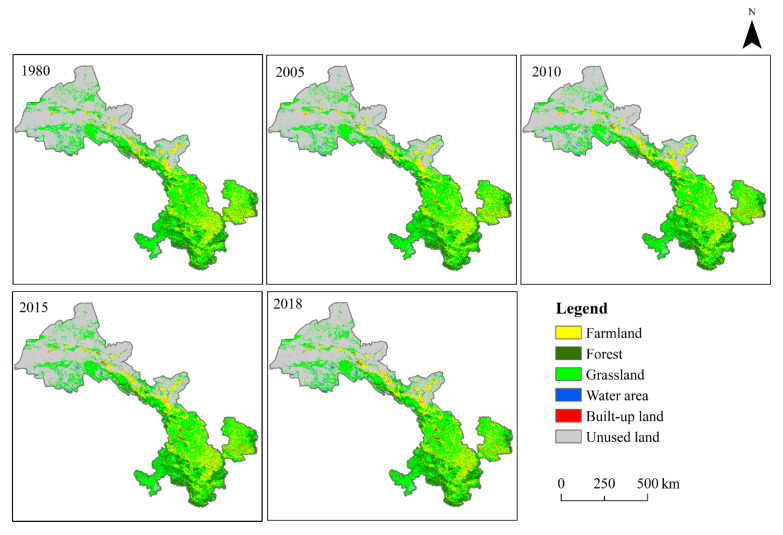
Land use and cover maps for 1980, 2005, 2010, 2015, and 2018.

LUCC raster data with 30 m resolution in 1980, 2005, 2010, 2015 and 2018 were obtained from the Resource and Environment Data Cloud Platform of the Chinese Academy of Sciences (http://www.resdc.cn), and reclassified into 6 types: (1) farmland: land for crop production; (2) forest: arbor forests and shrub forests; (3) grassland: artificial grassland and natural grassland; (4) built-up land: the land for urban areas, industrial or commercial zones and land for infrastructures; (5) water area: rivers, lakes, and reservoirs; and (6) unused land: barren land (Figure 2). These data were acquired from Landsat TM, ETM and OLI images using a visual interpretation method. The accuracy assessment of these data was conducted by previous studies based on human–computer interactive validation method and field survey, with a total accuracy > 90% [40,41]. The SRTM DEM (Shuttle Radar Topography Mission Digital Elevation Model) data with 30 m resolution produced by NASA were downloaded and used for calculation of aspect and slope based on GEE (Google Earth Engine), an online cloud platform for acquiring and processing remote sensing data. 

Socioeconomic data for each county in Gansu were acquired from the Gansu Statistical Yearbooks, including GDP (Gross Domestic Product), GDP per capita, agricultural outputs, industrial outputs, tertiary industry outputs, livestock number, and population change. Precipitation and temperature data were downloaded from the China Meteorological Data Service Center (http://data.cma.cn/en). Other vector data, including the administrative boundary, road, river system, and residential points of Gansu, were downloaded from the China National Geomatics Center (http://www.ngcc.cn/ngcc/). These data were spatialized and transformed into raster layers with a resolution of 30 m.

### 2.3. Methods

**Figure 3:**
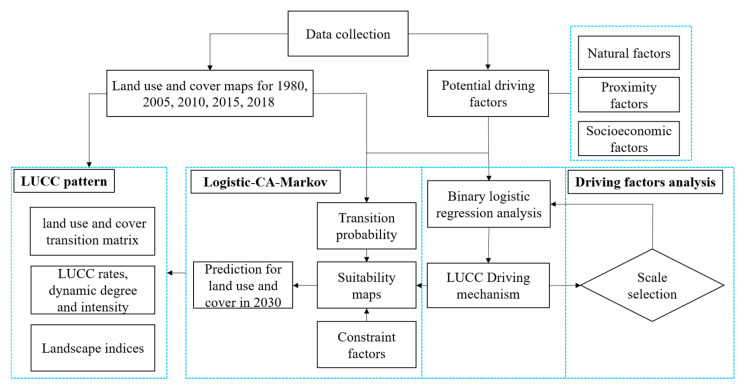
Framework building for the present study.

The framework of the present study (Figure 3) mainly includes the following four steps: (1) data collection, including land use and cover data for 1980, 2005, 2010, 2015 and 2018 and other related driving factors; (2) LUCC pattern analysis from 1980 to 2030, involving land use transition matrix analysis, LUCC’s rate and intensity analysis and landscape pattern analysis; (3) driving mechanism analysis from 1980 to 2018 based on a binary logistic regression model; and (4) prediction for future land use and cover by the integrated LCM model, which is based on historical land use and cover data for 2005, 2010, and 2015.

#### 2.3.1. Land Use and Land Cover Transition Matrix

The land use and land cover transition matrix [42] was used to present the conversion area between two stages in this study, which can be shown as follows:

(1) $$X=\left[\begin{array}{cccc} X_{11} & X_{12} & \ldots & X_{1j}\\ X_{21} & X_{22} & \ldots & X_{21}\\ \vdots & \ldots & \ddots & \vdots \\ X_{i1} & X_{i1} & \ldots & X_{ij} \end{array}\right]$$

where $X_{ij}$ is the land area of transition from land use and cover i to j.

#### 2.3.2. Dynamic Degree and Intensity of LUCC 

The land use dynamic degree index can reveal the quantity characters of certain land use change and represents the change rates of LUCC [43]. The equation is shown as follows:

(2) $$K=\frac{U_{bi}-U_{ai}}{U_{ai}}\times \frac{1}{T}\times 100\%$$

where $U_{ai}$ and $U_{bi}$ represents the area of land use i in the initial and the final year, respectively; and T is the time span between the initial and the final year.

The intensity index [43] can demonstrate the proportion of change areas of a certain land use in the whole research region, and can be calculated as:

(3) $$LTI_{i}=\frac{U_{bi}-U_{ai}}{LA}\times \frac{1}{T}\times 100\%$$

where $U_{bi}$ and $U_{ai}$ represents the certain land use area of the initial and the final year, respectively; T is the time span between the initial and the final year; and LA is the area of whole region.

#### 2.3.3. Logistic Regression Model 

We developed a binary logistic regression model to identify the dominant driving factors from 15 potential factors for each land use type in Gansu from 1980 to 2018 (Table 1). The model can be shown as follows [30]:

(4) $$logit\left(P\right)=\ln(\frac{P}{1-P})=\alpha +\sum_{i=1}^{n}\beta_{i}X_{i}$$

where P is the probability of the certain land use changes; $X_{i}$ is the selected driving factors; and $\beta_{i}$ is the regression coefficient of each selected variable.

**Figure 4:**
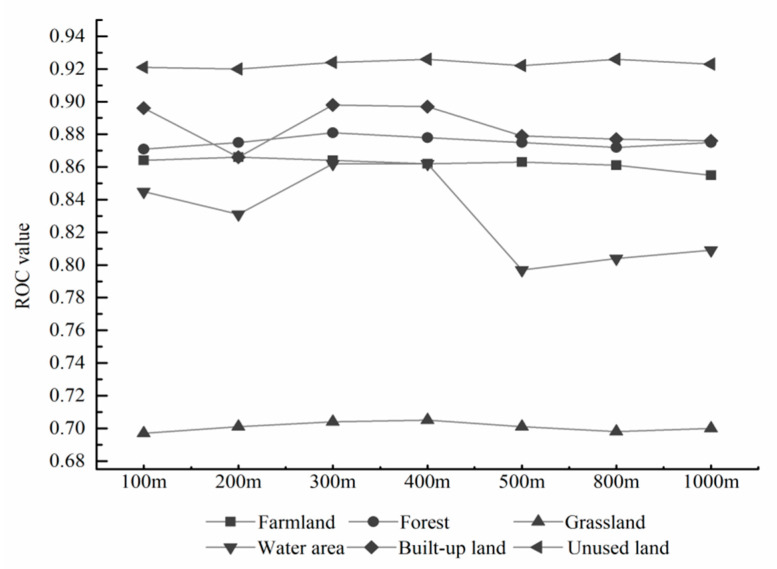
ROC value of each land use type in different modeling scales.

To avoid the spatial autocorrelation [44], we first randomly sampled 10,000 points within Gansu Province, to which the values of 15 raster layers were extracted in ArcGIS10.5, and then the attribute table of the point layer was exported as an Excel file for driving factors analysis. Finally, we used IBM SPSS to conduct the binary logistic regression analysis. The receiver operating characteristic (ROC) method was used to validate the result of binary logistic regression analysis [45]. Given that the spatial scale can greatly influence prediction accuracy, we compared the results of seven different scales (Figure 4). We found that the 300 m and 400 m were the relatively optimum scales without significant differences. But considering that higher resolutions can be better adapted to the analysis for land use types with small changes (such as the built-up lands) [16], we selected 300 m as the optimum modeling scale. 

#### 2.3.4. Integrated LCM Model

The LCM model integrates the logistic model and the CA-Markov model. The logistic model was firstly used to identify the driving factors of LUCC in Gansu, which is shown in previous section.

The CA-Markov model was used to predict the future LUCC maps. A Markov chain can effectively simulate the quantity of LUCC, but has limited ability to simulate the spatiotemporal characteristics of LUCC [19,46], whose equation can be shown as:

(5) $$s\left(t+1\right)=P_{ij}\times s\left(t\right)$$

where $P_{ij}$ is the transition probability between land use and cover type i and j; s(t) and s(t+1) are the state of land use and cover at period t and t+1, respectively.

The CA model has adequate capability of simulating spatiotemporal change of land use and cover by defining certain land use transition rules, thus can be integrated with the Markov model to simulate the future LUCC [47]. The model can be defined by the following equation:

(6) $$s_{ij}^{t+1}=f\left(s_{ij}^{t},\;Q_{ij}^{t},V\right)$$

where t and t+1 the initial and final time of the simulation; $s_{ij}^{t}$ and $s_{ij}^{t+1}$ is the state of the cell in row i and column j at time t and t+1, respectively; $Q_{ij}^{t}$ is the state of neighbors of the cell in row i and column j at time t*;* V is the suitability factors set; and f is the transition rule function. 

Specifically, the processes of LCM in the present study can be divided into three steps: (1) using a binary logistic regression model to analyze the driving factors; (2) including results from driving factors analysis in the MCE process to create suitability maps, with the help of the coefficient of variation (CV) to assign weights for factors; and (3) predicting LUCC spatiotemporal pattern in the future by using a CA model.

Suitability factor maps can be used to predict the land use and cover change in the future more accurately. In this research, we created suitability maps according to the following steps: Firstly, we selected suitability factors for each land use type according to the logistic analysis results and standardized those factors. Secondly, we determined the weights of driving factors using the CV method [48] (Table 2). Finally, we created the suitability maps of each land use and cover type by the MCE model in IDRISI17.2 software.

We set two prediction scenarios for the LUCC of Gansu Province. Scenario 1, historical change trend: the land use and cover would change according to the historical trend; Scenario 2, strict environmental protection: the farmlands with slope lower than 25°, forests and grasslands would be protected from transiting to other lands. We achieved these two scenarios by setting the constraint factors in MCE model. Specifically, there was no limit in Scenario 1. As the farmlands with slope lower than 25° in Scenario 2, the total forests and grasslands were set as constraint factors which were not involved in the prediction process. 

According to the general planning of the Grain for Green Project (GGP) of Gansu Province, the second stage of GGP (2014–2020) is coming to an end by 2020. Here we predicted the land use and cover change in 2030 under different scenarios to provide some references and guidance for government planning in the next 10 years (from 2020 to 2030). Besides, the CA-Markov model was used to predict the land use and cover based on historical LUCC data (2005, 2010, and 2015 in this study). The prediction period in this model should be the integral multiple of interval of historical data (5 years in this study), i.e., 5 years, 10 years, 15 years, and so on. So, in theory, we could predict the land use in 2020, 2025 and 2030, and so on. However, considering the first point, we finally chose to predict the land use condition in 2030 rather than 2020 or 2025.

#### 2.3.5. Model Validation

The kappa index was used to validate the accuracy of the results of prediction, which is calculated from the difference between the actual land use state and the prediction land use types [49]. It can be computed by the following equation:

(7) $$K=(P_{a}-P_{e})/(P_{i}-P_{e})$$

where *K* is the kappa index; $P_{a}$ is the actual accuracy; $P_{e}$ is the expected prediction accuracy; and $P_{i}$ is the ideal accuracy (100%). This index can be calculated using the validate module of the IDRISI17.2 software. 

#### 2.3.6. Landscape Patterns Analysis

Landscape index can be used to describe landscape pattern changes [50]. In the present study, four groups of landscape indices were selected, embracing area-edge indices, shape indices, landscape aggregation indices, and landscape diversity indices. Specifically, area-edge indices described the size of patches and the amount of edge, including largest patch index (LPI) and edge density (ED); shape indices included landscape shape index (LSI) and area wight mean shape index (AWMSI), which indicates the overall geometric complexity of landscape patch; landscape aggregation indices included contagion and cohesion, indicating tendency of patch types to be spatially aggregated; diversity indices, such as Shannon’s diversity index (SHDI) and Shannon’s evenness index (SHEI), represent landscape richness and evenness. FRAGSTATS 4.2 was used to calculate all these indices [51].

## 3. Results

### 3.1. LUCC Pattern from 1980 to 2018

Between 1980 and 2005, a total area of 10,677.4 km^2^ experienced changes, which accounted for 2.5% of the total area of Gansu (Table 3). Among all the six land use types, the farmlands, the forests, and the built-up lands had experienced a net increase of 853.24 km^2^, 204.29 km^2,^ and 485.97 km^2^, respectively. Meanwhile, the grasslands, water areas, and the unused lands decreased by 377.64 km^2^, 186.92 km^2^, and 978.94 km^2^, respectively. Specifically, the increases of farmlands were mainly derived from reclaiming the grasslands and the water area, and the increased forests were converted from the grasslands and the farmlands, while the built-up lands mostly increased from occupying the farmlands and the grasslands. From 2005 to 2018, the total area that underwent changes was about 58065.09 km^2^, which was 5.5 times as large as that in the first stage (Table 4). The farmlands and the unused lands decreased by 1521.38 km^2^ and 1741.67 km^2^, respectively, but other land types increased, suggesting that the decreases of farmlands and the unused lands were responsible for the increases of the four other land use types.

The LUCC’s rates from 1980 to 2018 in Gansu Province are presented in Table 5. In terms of the LUCC dynamic degree, the built-up lands and the forests increased at an annual rate of 0.569% and 0.02% between 1980 and 2005, respectively. From 2005 to 2018, they continued to increase at an annual rate of 3.03% and 0.065%, respectively. However, the unused lands declined during the whole period, at an annual rate of 0.022% and 0.076% in the first period and the second period, respectively. The farmlands increased by 0.051% per year between 1980 to 2015 but decreased annually by 0.168% from 2005 to 2018. The water areas and the grasslands decreased at an annual rate of 0.205% and 0.01%, respectively, at the first stage, however, they surged into the second stage with an annual rate of 1.146% and 0.034%, respectively. As for the LUCC intensity, all land use types underwent a drastic increase from 1980 to 2018 except the unused lands. Among all these land use types, the change intensity of built-up land was the most significant, having increased from 0.004% in the first period to 0.029% in the second stage. The unused lands and the farmlands ranked second to the built-up land. Three other land use and cover types only changed slightly.

### 3.2. Driving Factors of LUCC 

Table 6 shows the logistic regression analysis results. The Wald statistic indicated the cumulative contributions of indicators to dependent variables. The Exp (B) column contains the odds ratios of land use changes occurring, representing the influencing intensity of each factor. 

We found that only three common factors influenced all six land use types: they are elevation, precipitation, and distance to residential points. The Wald statistic indicated that the total contribution rates of these three variables was 672.37, 592.40, 169.18, 61.64, 84.21, and 1130.73 for the farmlands, forests, grasslands, water area, built-up lands, and the unused lands, respectively. Additionally, the land use changes were also affected by other factors. For example, slope and GDP per capita were the other main factors affecting the farmlands changes, with contribution rates of 160.53 and 94.71. The contribution rates of the remaining factors were 155.10, which included aspect, industrial outputs, distance to roads, distance to water, agricultural outputs, population growth rates, and livestock numbers. The forests were also affected by another seven factors, including aspect, slope, temperature, GDP growth, GDP per capita, population growth, as well as livestock numbers, with a total contribution rate of 331.45. Among them, GDP per capita, slope, and temperature influenced the forests’ changes the most. The grasslands were also influenced by aspect, slope, temperature, GDP growth, tertiary industry output, industrial output, distance to roads, GDP per capita, population growth, and livestock numbers, in addition to three common factors, with a total contribution rate of 195.23. The changes to the water area were also driven by slope, temperature, industrial output, distance to road, distance to water, and GDP per capita, with a contribution rate of 99.38. Distance to water affected water area changes the most with a contribution rate of 44.08. The built-up land changes were mainly influenced by slope, with a contribution rate of 53.99. Besides, distance to road and agricultural outputs influenced these changes as well, with a contribution rate of 52.16. The changes to unused land were related to temperature, tertiary industry output, industrial output, distance to road, distance to water, GDP per capita, and livestock numbers, with a total contribution rate of 550.81.

### 3.3. Model Validation

**Figure 5:**
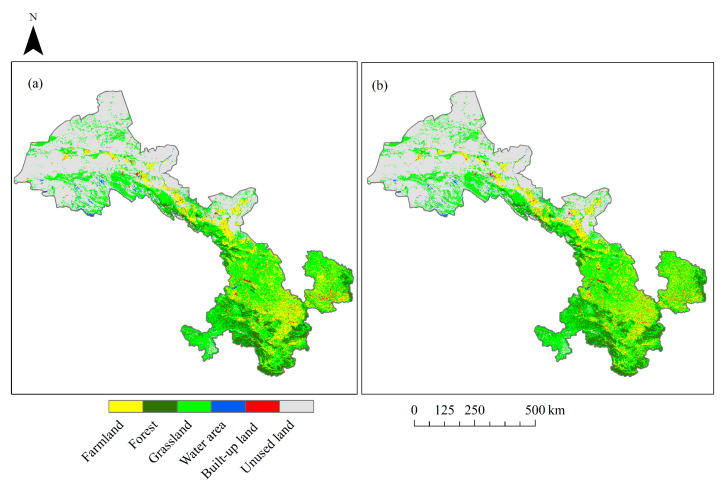
Predicted and actual land use and cover maps in 2015: (**a**) predicted land use maps by model; (**b**) actual maps from remote sensing.

For validating the reliability of this projection method, the land use and cover in 2015 was predicted by the LCM model based on historical land use and cover maps of 2005 and 2010, and then compared with the actual land use and cover maps in 2015 (Figure 5). Spatially, there were no significant differences in spatial pattern between the predicted land use maps and the actual one. The total kappa index was 0.92, manifesting that this model and comprehensive suitability maps can be well adapted to predict the land use and cover conditions in the future. Comparing further the difference in each land use type between predicted and actual land use (Table 7), we found that the total error was 0.01%, which was an ideal result. The predicted land use and cover was generally overestimated for all land cover types except for the unused land, with more error specifically for water areas and built-up lands (with an error of 60% and 37%). Therefore, we can say that predicting future land use and cover conditions following this method is reliable and acceptable in general.

### 3.4. Prediction for Future LUCC Under Two Scenarios 

**Figure 6:**
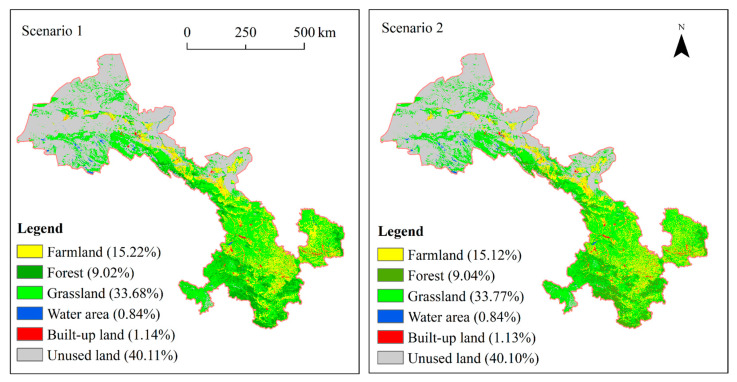
Prediction for land use and cover in 2030 under two scenarios.

Using the LCM model, we predicted the land use pattern in 2030 under two different prediction scenarios. There would be no significant difference in the spatial allocation pattern of land use and cover between the two scenarios (Figure 6). The farmlands would be mainly located in the oases of the Hexi Corridor in West Gansu Province and the Loess hilly region in the east, covering an area of about 64619.53 km^2^ in Scenario 1 and 64198.53 km^2^ Scenario 2. The grasslands and forests with an area of 181,307.40 km^2^ and 181,793.70 km^2^ in Scenario 1 and 2, respectively, would mainly cover the Qilian mountain area in Middle Gansu and the Qinling mountain area in the south part, as well as the Qinghai–Tibet plateau in the southwest. The water area would sporadically distribute in the marginal area of Qinghai–Tibet plateau in Northwest Gansu Province, with an area of 4832 km^2^ and 4808.93 km^2^ in Scenario 1 and 2 respectively, most of which are plateau or alpine lakes. The built-up land in Scenario 1 and 2 would occupy an area of 4832 km^2^ and 4808.93 km^2^, respectively, dispersedly located in the flat valley area suitable for living and development. The unused land would cover an area of 170,320.57 km^2^ in Scenario 1 and 170,293.29 km^2^ in scenario 2, respectively, covering a large portion of West Gansu Province. 

Compared with Scenario 1, the farmlands, built-up lands, and the unused lands would decrease by 0.1%, 0.01%, and 0.01%, respectively, the forests and grasslands would increase by 0.02% and 0.09%, and the water areas would remain unchanged in Scenario 2. The area of the farmlands will be smaller in Scenario 2 than in Scenario 1, while the spatial distribution of the farmlands will be more optimized because of environmental management measures in Scenario 2.

### 3.5. Landscape Pattern Change from 1980 to 2030

**Figure 7:**
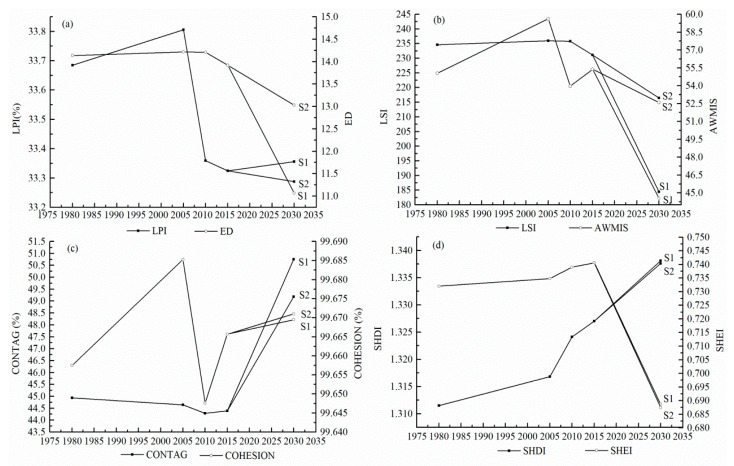
Landscape indices in Gansu Province: (**a**) area-edge indices; (**b**) shape indices; (**c**) landscape aggregation indices; (**d**) landscape diversity indices.

Figure 7 shows the change of landscape indices from 1980 to 2030. The years of 2005 and 2015 were two turning time-points, the landscapes showed different change patterns before and after them.

Generally, the eight landscape indices experienced a drastic change during the first period (1980 to 2015). In particular, the LPI, ED, LSI, and AWMIS indices increased before 2005 and then declined during the next 10 years. CONTAG increased before 2005 and then began to decrease and increased again after 2010. COHESION declined continuously in the period between 1980 and 2010, and then rose slightly after 2010. SHDI and SHEI increased persistently over the 35 years until 2015. 

After 2015, most of the landscape indices would show consistent tendencies under two future scenarios but would be different in change rates. ED, LSI, AWMSI and SHEI would show a trend of decrease in both scenarios. Meanwhile, CONTAG, COHESION and SHDI show an increasing trend. However, LPI increases in Scenario 1 but decreases in Scenario 2. Comparing the indices in 2030 under two scenarios, we found that the indices that are greater in scenario 2 included the ED, LSI, AWMSI, COHESION, and the indices that are greater in Scenario 1 embraced the LPI, CONTAG, SHDI, and SHEI.

## 4. Discussion

### 4.1. Spatiotemporal Characteristics of LUCC from 1980 to 2018

Generally, the spatiotemporal characteristics of LUCC in Gansu Province presented differences in different stages. In the first stage (1980–2005), land use and cover transition structure was not very favorable to the sustainability of the environment and socio-economy, and large amounts of grasslands, water areas, and unused lands were used to cultivate crops, expand urban areas and build roads, resulting in ecological problems, such as sandstorms and desertification [52,53,54]. Meanwhile, the forests increased as a results of environmental protection policy [4,39] (such as the Three-North Forest Shelterbelts Program launched in 1979), however it was mainly based on destroying grasslands and farmlands and just a little area of unused lands were converted (Table 3). During the second period (2005–2018), the land use structure tended to be more rational. For instance, forests, grasslands, and the water areas were protected and increased to a large extent (Table 4), which mainly benefited from the Grain for Green Project (GGP) launched in 1999 by the Chinese government [20,55]. The urban expansion mainly occupied the unused lands rather than lands with more ecological benefits, such as the forests and the grasslands. As a result, the ecological environment took a turn for the better, the sandstorms and desertification were kept within limits in this stage [56].

The land use dynamic degree and intensity can clearly indicate the rates of area changed of each land use type and strengthen the comparability between different land use and cover types [57]. In this present study, we found that the LUCC dynamic degree and intensity in the second stage (2005 to 2018) were significantly greater than those in the first stage (1980 to 2005) in Gansu Province, which demonstrated a more extensive LUCC between 2005 and 2018 in this region. A similar result was reported by Jin et al [58]. This was due to the rapid socioeconomic improvements, as well as the implementation of the GGP during this period. Specifically, the built-up lands had the greatest dynamic degree both in the first and the second stage among these six land use types, and their area increased all the time, which may be attributed to rapid urbanization in this area since the implementation of the Reform and Opening up Policy in 1978 [5,18]. The change of speed of unused lands was relatively slow, but the changed area was very large and accounted for a high percentage of the total area of the Gansu Province. Therefore, it had the greatest LUCC intensity during the whole period. In addition, the changes in dynamic degree and intensity of farmlands, forests, the grasslands, as well as the water area were directly subjected to the ecological protection project, which significantly accelerated the process of farmland returning to green lands and vegetation recovery [59].

The above analysis suggests that the land use and cover in Gansu Province underwent great changes over the past decades, which is similar to research results from other arid and semiarid area, such as the Taperoá River basin in Northeastern Brazil [26], the north-western coastal desert of Egypt [60] and the Middle Suluh Valley in Northern Ethiopia [14]. Moreover, we found that environmental protection policy contributed to the vegetation recovery in the Middle Suluh Valley, Northern Ethiopia [14], which is also consistent with our findings in Gansu Province in this study. 

### 4.2. Driving Mechanism of LUCC 

Generally, LUCC is the result of the comprehensive influences of so many complex and diverse factors [17,61,62]. Previous studies have demonstrated that on a global scale, human-driven changes in land use and cover accounted for most of the changes of land surface. However, the main factors vary according to the nature and extent of the area [63]. In this study, we analyzed the driving factors of LUCC in Gansu Province from 1980 to 2018 from three angles: natural factors, proximity factors, and socioeconomic factors, finding that natural factors were dominant in LUCC in Gansu Province during this period, which was different from the other researches [14,16,17,64,65].

#### 4.2.1. Natural Factors

Natural factors are generally the material foundations and environmental conditions of LUCC, including terrain and climate, which establish the basic pattern of LUCC [62]. Some researchers thought that natural condition is not one of the essential factors influencing LUCC in the short term, especially in those regions with rapid urbanization and expanding population [17,65]. In our study, however, it was found that natural factors played fundamental roles in the LUCC of Gansu from 1980 to 2018. According to the Wald statistics in Table 6, among all these six natural factors, elevation, precipitation, and slope were the dominant ones, which deeply affected the changes of the farmlands, forests, grasslands, built-up lands, as well as the unused lands. This is because Gansu is an inland Province located in the junctional zone of the Qinghai–Tibetan plateau, the Loess plateau, and the Inner Mongolian plateau with arid climates and complex terrains. For example, in these complex conditions, the farmlands and residential areas must be distributed in areas with a slighter slope, lower altitude, and more precipitation to avoid natural disaster, as well as water and soil loss [66,67]. On the other hand, the unused lands, and some farmlands in the areas with high elevations and steep slopes are covered with planted trees and grasses because of GGP [14,16].

#### 4.2.2. Proximity Factors

Proximity factors were regarded as nonnegligible factors influencing LUCC, which directly represent the intensity of effects of human activities and can influence the LUCC process profoundly [67,68]. Li et al. [66] found that the farmlands in the Yellow River Delta in the last 30 years were influenced by the distance to coastline, distance to cities, and distance to rivers. Research by Liu et al. [68] suggested that the urban expansion in central Liaoning urban agglomerations was affected by distance to rivers, distance to residential areas, as well as distance to roads. In this study, we also found the relationships between the LUCC and these proximity factors. With the distance to residential points, rivers and roads increasing, their effects on the farmlands, forests, grasslands, water areas, and the built-up lands weakened gradually, which indicated that land use and cover surrounding human activities was more easily influenced [69]. Besides, the changes of built-up lands showed a significantly positive relationship with distance to human activities. This was because the unused land was originally far away from humans in Gansu. But it did not mean that the changes of unused lands had nothing to do with human activities. In fact, on the contrary, a large area of unused lands far from humans was covered with planted grasses and trees in this area [39].

#### 4.2.3. Socioeconomic Factors

Socioeconomic factors played dominant roles in driving LUCC to a large extent, especially in some countries or regions where the economy is rapidly developing, the population is increasing, the institutions are reforming [70]. However, in this study, we discovered that compared with natural and proximity factors, the effects of socioeconomic factors on LUCC in Gansu Province were not obvious. This may be attributed to the relatively slow socioeconomic development of Gansu Province, including the lower urbanization rates and industrialization rates [59]. Limited by a fragile ecological environment, a less competitive investment environment, and inconvenient traffic conditions, Gansu was one of the most undeveloped provinces in China [71]. As a result, its socio-economy did not have enough capacity to drive intensive LUCC. Among these socioeconomic factors, GDP, GDP per capita, population, livestock, and industrial output affected LUCC the most. Specifically, the farmlands and grasslands were affected by socioeconomic factors the most; this was mainly because increasing food demands required more lands for cultivating crops, and expansion of industrial lands resulted in the occupying of grasslands [59].

### 4.3. Land Use and Cover in 2030

According to prediction results for 2030 under two scenarios by the LCM model, we can find that the land use pattern under strict ecological protection scenarios would tend to be more reasonable than under the historical trend scenario, as reported by other studies [21,72]. Specifically, the area of forests and grasslands would be larger, and more unused land would be used for green lands under the environmental protection scenario. Besides, although the area of farmlands is smaller in the historical trend scenario, their allocation would be more reasonable, as the farmlands unsuitable for cultivation would be used to plant trees and grasses. In addition, the built-up lands area under the ecological protection scenario would be less than it would be under the historical trend scenario, suggesting that the unreasonable expansion of urban areas would be confined [73].

It is widely believed that the best measures to increase green lands is to transfer wasteland and unused land with good hydrological conditions into ecological land and expand existing forests and grasslands [73]. However, Gansu is a typical arid and semiarid area with a harsh natural environment. After undergoing decades of environment recovery, its potential for large-scale vegetation recovery would be limited to a great extent in the future. Therefore, the best way to increase ecological land use is to protect existing forests and grasslands from being transformed into other lands, rather than implementing ecological engineering continuedly (such as GGP) in following years. 

### 4.4. The Changes of Landscapes 

Landscape pattern change analysis is of great significance to realizing the land use change process and its driving forces. Over the past 35 years, up to 2015, the landscape underwent great changes in Gansu Province. From the changes of area-edge indices, we found that LPI and ED experienced a decrease in general, suggesting that the area of dominant landscape decreased, and the landscape pattern tended to be more coherent. The decreased shape index indicated that the landscape became more regular and not complicated. As for the aggregation indices, the decreased CONTAG indicated that the agglomeration of landscape patch was declined; meanwhile, the fluctuated COHESION suggested that the boundary of adjacent landscapes was not stable. The increased SHDI and SHEI indicated that the landscape distributions became more diversified and spatially balanced. Overall, the landscape pattern in Gansu Province during this period tended to be more optimized, which demonstrated that the ecological lands experienced a recovery in Gansu during this stage, as the result of many years of environment recovery [20]. 

In 2030, the landscape pattern would show differences under two prediction scenarios. In terms of area-edge indices, the discrepancy in LPI and ED between Scenario 1 and 2 indicate that the landscape pattern would be more cracked under the ecological protection scenario, but it would contribute to edge effects, which is significant to biological diversity [74]. The differences in shape indices between the two prediction scenarios suggests that the environmental protection strategy would make the landscape shape more complex and irregular; it can also be beneficial to the edge effects of landscape. Meanwhile, the aggregation indices for 2030 indicate that the homogeneous landscapes would be more spatially scattered and uniform, but different landscapes would congregate and become closely connected. Besides, we find that the SHDI and SHEI under historical trend scenarios would be slightly greater than those in the environmental protection scenario, but the differences between them are not significant. This suggests that the landscape type diversity under both scenarios would be similar. Overall, the landscape pattern would tend to be more reasonable under the strict environmental protection situation. Similar results were reported in previous studies [21,68,75].

### 4.5. Availability of the Integrated LCM Model

Validation for the integrated LCM model showed that using this model to predict the LUCC of Gansu Province in the future is generally reliable. However, for each land use type, there existed a difference in prediction accuracy. The area of predicted farmlands was larger than the observed area. This was due to the fact that given the basic farmlands protection policy, we set the farmlands located at a slight slope as a constraint factor in the suitability maps, and they would be protected and not converted to other lands in the predicted condition. But in an actual situation, this policy may not be implemented thoroughly, and land reclamation may occur all the time [76]. In addition, the prediction accuracy of the water areas and the built-up lands was unsatisfying, which resulted from the model’s inherent defect of underperforming in more delicate simulations [25,27]. The changed area of water areas and built-up lands only accounted for a small proportion of the total area of Gansu, which made the prediction process model uncertain [25]. Especially as the water area is also easily influenced by precipitation and evaporation and is unstable in arid and semiarid regions, making the prediction process more occasional [25]. This result was similar to the research on prediction for water area in a Zhangye oasis [20].

### 4.6. Implication for Optimizing the Land Use and Cover in Global Arid and Semiarid Areas

Based on the results of this case study, some suggestions can be made to instruct the land use and management of arid and semiarid regions. Firstly, moderate policy interference by government can be beneficial to increase green land and optimize land use. Therefore, we recommend that environmental management policies applied to local conditions should be formulated by the government to prevent unreasonable land use transition in arid and semiarid areas, such as large-scale transformations from green lands to built-up lands and farmlands. Secondly, for arid and semiarid areas, the best way to increase green lands is to protect existing vegetation rather than planting more trees and grasses. As these regions are generally constrained by water resources, the new developments of vegetation may not perform well. Thirdly, this work, as well as many previous studies, has demonstrated that the remote sensing technique and relevant models (such as the LCM model in this present study) can be well applied to monitoring and modelling the LUCC in arid and semiarid regions. Consequently, using these methods to monitor and predict the LUCC will be significant to optimizing land use and cover and formulating feasible land use and plan policies in arid and semiarid area.

## 5. Conclusions 

In this research, we analyzed the LUCC pattern from 1980 to 2018 in Gansu Province and revealed its driving mechanism based on a binary logistic regression model. Then, taking the driving forces and some environment policies into account, we predicted the future land use and cover conditions in 2030 under different scenarios, using the CA-Markov model. We found that the LUCC spatiotemporal patterns in Gansu Province tended to be more favorable from 1980 to 2018 and ecological lands were well protected from reclaiming, and thus recovered gradually. In general, natural factors were the main force affecting the changes in land use and cover in Gansu during this time. However, the effects of socioeconomic factors on LUCC were not significant. Landscape pattern analysis indicated that predicted land use and cover in 2030 under the ecological protection scenario would be more rational than under the historical trend scenario. Application of the integrated LCM model to predict LUCC of Gansu in the future was reliable.

This current study provides a basic framework for the application of the integrated logistic-CA-Markov model to detect LUCC in arid and semiarid regions. However, limited by the inherent defects of the simulation model we adopted, the prediction for water areas and built-up land was not very accurate. Thus, some improvements for the integrated model are needed in future research.

## Figures and Tables

**Table 1:** Potential driving factors.

Factor Types	Potential Driving Factors	Description	Units	Signs
Natural factors	temperature	annual mean temperature	mm	X1
	precipitation	annual mean precipitation	°C	X2
	elevation	DEM data	m	X3
	aspect	range from 0 to 360	°	X4
	slope	range from 0 to 90	°	X5
Proximity factors	distance to water body	Euclidean distance to water body	km	X6
	distance to road	Euclidean distance to road	km	X7
	distance to residential point	Euclidean distance to residential point	km	X8
Socioeconomic factors	GDP change	mean annual growth rate of GDP	%	X9
	GDP per capita	mean annual growth rate of GDP per capita	%	X10
	agricultural outputs	mean annual growth rate of agricultural output	%	X11
	industrial outputs	mean annual growth rate of industrial output	%	X12
	tertiary industry outputs	mean annual growth rate of tertiary industry output	%	X13
	livestock number	mean annual growth rate of livestock	%	X14
	population change	mean annual natural population growth rate	%	X15

**Table 2:** Weights of selected driving factors.

Potential Factors	Farmland	Forest	Grassland	Water Area	Built-Up Land	Unused Land
X1	-	0.039	0.028	0.034	-	0.033
X2	0.077	0.113	0.082	0.098	0.164	0.096
X3	0.044	0.064	0.046	0.055	0.093	0.054
X4	0.067	0.098	0.071	-	-	-
X5	0.114	0.166	0.121	0.144	0.242	0.170
X6	0.138	-	-	0.174	-	0.135
X7	0.109	-	0.116	0.138	0.232	0.117
X8	0.095	0.139	0.101	0.120	0.202	-
X9	-	0.084	0.061	-	-	-
X10	0.066	0.096	0.070	0.083	-	0.081
X11	0.031	-	-	-	0.066	-
X12	0.121	-	0.128	0.153	-	0.149
X13	-	-	0.029	-	-	0.034
X14	0.106	0.155	0.112	-	-	0.131
X15	0.032	0.047	0.034	-	-	-

NOTE: “-” represents that this factor was not selected.

**Table 3:** Land use and cover conversion matrix from 1980 to 2005 (km^2^).

Land Use and Cover Types in 1980	Land Use and Cover Types in 2005						Total
	Farmland	Forest	Grassland	Water Area	Built-Up Land	Unused Land	
Farmland	-	341.11	1997.17	46.03	479.05	136.66	3000.02
Forest	211.59	-	918.90	6.96	19.08	26.39	1182.90
Grassland	2373.87	983.81	-	34.55	71.69	674.99	4138.90
Water area	165.07	22.30	84.72	-	8.59	58.49	339.17
Built-up land	105.53	4.83	25.23	1.52	-	1.92	139.02
Unused land	997.22	35.15	735.23	63.19	46.60	-	1877.38
Total	3853.27	1387.19	3761.25	152.25	625.00	898.44.	10677.41

**Table 4:** Land use and cover conversion matrix from 2005 to 2018 (km^2^).

Land Use and Cover Types in 2005	Land Use and Cover Types in 2018						Total
	Farmland	Forest	Grassland	Water Area	Built-Up Land	Unused Land	
Farmland	-	1335.04	12703.67	319.27	1916.13	632.04	16906.15
Forest	1121.55	-	5412.95	57.88	80.93	327.87	7001.17
Grassland	11532.78	5624.00	-	332.93	626.79	5367.83	23484.34
Water area	270.05	56.21	272.08	-	43.77	205.70	847.81
Built-up land	1119.76	51.28	295.43	24.19	-	29.94	1520.59
Unused land	1340.63	312.00	5535.72	664.38	452.30	-	8305.03
Total	15384.77	7378.53	24219.85	1398.66	3119.92	6563.37	58065.09

**Table 5:** Dynamic degree and intensity of LUCC from 1980 to 2018.

Land Use and Cover Types	Dynamic Degree (%)		Intensity (%)	
	1980–2005	2005–2018	1980–2005	2005–2018
Farmland	0.051	−0.168	0.008	−0.028
Forest	0.020	0.065	0.002	0.006
Grassland	−0.010	0.034	−0.003	0.012
Water area	−0.205	1.146	−0.002	0.010
Built-up land	0.569	3.030	0.004	0.029
Unused land	−0.022	−0.076	−0.009	−0.033

**Table 6:** Results of binary logistic regression analysis.

Land Use and Cover Types	Driving Factors	Regression Coefficients	Standard Error	Wald Statistic	Significance Level	Exp (B)
Farmland	X4	−0.001	0.000	10.281	0.001	0.999
	X3	−0.001	0.000	170.418	0.000	0.999
	X2	0.003	0.000	138.424	0.000	1.003
	X5	−0.052	0.004	160.534	0.000	0.949
	X12	−2.723	0.309	77.781	0.000	0.066
	X7	−0.065	0.000	13.307	0.000	1.000
	X8	−0.206	0.000	363.532	0.000	1.000
	X6	−0.051	0.000	11.809	0.001	1.000
	X11	6.017	1.053	32.631	0.000	410.209
	X10	5.910	0.607	94.715	0.000	368.881
	X15	−0.536	0.253	4.510	0.034	0.585
	X14	0.193	0.088	4.788	0.029	1.212
Forest	X4	0.001	0.000	3.967	0.046	1.001
	X3	0.001	0.000	200.220	0.000	1.001
	X2	0.005	0.000	380.118	0.000	1.005
	X5	0.049	0.004	163.644	0.000	1.050
	X1	0.160	0.021	56.974	0.000	1.173
	X9	−2.280	0.656	12.079	0.001	0.102
	X8	−0.030	0.000	12.065	0.001	1.000
	X10	5.248	0.685	58.726	0.000	190.262
	X15	−0.755	0.308	5.998	0.014	0.470
	X14	−0.512	0.093	30.063	0.000	0.600
Grassland	X4	0.000	0.000	5.913	0.015	1.000
	X3	0.000	0.000	71.275	0.000	1.000
	X2	0.001	0.000	54.098	0.000	1.001
	X5	0.008	0.003	10.756	0.001	1.009
	X1	−0.041	0.014	8.750	0.003	0.960
	X9	−2.202	0.496	19.749	0.000	0.111
	X13	0.965	0.400	5.834	0.016	2.625
	X12	−1.196	0.245	23.870	0.000	0.302
	X7	−0.017	0.000	6.290	0.012	1.000
	X8	−0.020	0.000	43.804	0.000	1.000
	X10	3.961	0.491	64.958	0.000	52.494
	X15	0.945	0.171	30.683	0.000	2.573
	X14	−0.326	0.076	18.425	0.000	0.722
Water area	X3	0.001	0.000	37.197	0.000	1.001
	X2	−0.002	0.001	6.110	0.013	0.998
	X5	−0.046	0.013	11.821	0.001	0.955
	X1	0.195	0.069	8.047	0.005	1.215
	X12	−4.478	1.274	12.356	0.000	0.011
	X7	−0.129	0.000	16.105	0.000	1.000
	X8	−0.102	0.000	18.335	0.000	1.000
	X6	−0.001	0.000	44.078	0.000	0.999
	X10	5.070	1.919	6.979	0.008	159.219
Built-up land	X3	0.000	0.000	5.749	0.016	1.000
	X2	0.003	0.001	31.395	0.000	1.003
	X5	−0.139	0.019	53.999	0.000	0.870
	X7	−0.390	0.000	19.492	0.000	1.000
	X8	−0.255	0.000	47.063	0.000	1.000
	X11	−6.555	2.902	5.101	0.024	0.001
Unused land	X3	0.000	0.000	108.017	0.000	1.000
	X2	−0.012	0.000	933.409	0.000	0.988
	X1	−0.562	0.036	240.324	0.000	0.570
	X13	1.762	0.584	9.110	0.003	5.825
	X12	4.198	0.422	98.789	0.000	66.535
	X7	0.044	0.000	25.954	0.000	1.000
	X8	0.035	0.000	89.307	0.000	1.000
	X6	0.081	0.000	76.450	0.000	1.000
	X10	−6.935	0.808	73.715	0.000	0.001
	X14	−1.608	0.312	26.473	0.000	0.200

**Table 7:** Validation of predicted land use and cover in 2015 (km^2^).

Land Use and Cover	Farmland	Forest	Grassland	Water Area	Built-Up Land	Unused Land	Total
Actuality	64927.88	38226.20	143,281.66	3357.43	4260.18	171,445.61	425,498.98
Prediction	72139.68	38431.24	145,349.78	5376.15	5849.64	158,393.94	425,540.43
Error	0.111	0.005	0.014	0.60	0.373	−0.076	0.0001
